# Combining Molecular Dynamics and Docking Simulations to Develop Targeted Protocols for Performing Optimized Virtual Screening Campaigns on the hTRPM8 Channel

**DOI:** 10.3390/ijms21072265

**Published:** 2020-03-25

**Authors:** Carmine Talarico, Silvia Gervasoni, Candida Manelfi, Alessandro Pedretti, Giulio Vistoli, Andrea R. Beccari

**Affiliations:** 1 Dompé Farmaceutici SpA, Via Campo di Pile, 67100 L’Aquila, Italy; carmine.talarico@dompe.com (C.T.); candida.manelfi@dompe.com (C.M.); 2 Dipartimento di Scienze Farmaceutiche, Università degli Studi di Milano, Via Mangiagalli, 25, I-20133 Milano, Italy; silvia.gervasoni@unimi.it (S.G.); alessandro.pedretti@unimi.it (A.P.); giulio.vistoli@unimi.it (G.V.)

**Keywords:** TRPM8, docking simulations, structure-based virtual screening, multiple receptor conformations, consensus approaches, LiGen™, PLANTS, EFO

## Abstract

**Background**: There is an increasing interest in TRPM8 ligands of medicinal interest, the rational design of which can be nowadays supported by structure-based in silico studies based on the recently resolved TRPM8 structures. **Methods**: The study involves the generation of a reliable hTRPM8 homology model, the reliability of which was assessed by a 1.0 μs MD simulation which was also used to generate multiple receptor conformations for the following structure-based virtual screening (VS) campaigns; docking simulations utilized different programs and involved all monomers of the selected frames; the so computed docking scores were combined by consensus approaches based on the EFO algorithm. **Results**: The obtained models revealed very satisfactory performances; LiGen™ provided the best results among the tested docking programs; the combination of docking results from the four monomers elicited a markedly beneficial effect on the computed consensus models. **Conclusions**: The generated hTRPM8 model appears to be amenable for successful structure-based VS studies; cross-talk modulating effects between interacting monomers on the binding sites can be accounted for by combining docking simulations as performed on all the monomers; this strategy can have general applicability for docking simulations involving quaternary protein structures with multiple identical binding pockets.

## 1. Introduction

TRPM8 is a tetrameric nonselective cation channel, which is primarily activated by cold through a multimodal mechanism also influenced by other factors, such as voltage, pH plus some specific ligands (e.g., menthol) [1,2,3]. TRPM8 is mainly expressed in peripheral sensory neurons but is also present in the prostate, bronchopulmonary tissue, bladder, and the urogenital tract [4]. Starting from its key implication in pain conditions such as cold allodynia after inflammation [5], TRPM8 is nowadays known to be involved in various pathological conditions ranging from tumor progression [6] to migraine [7] or dry eye disease [8]. Not to mention that recent studies revealed a TRPM8 role also in irritable bowel syndrome [9], oropharyngeal dysphagia and chronic cough [10]. 

Along with the binding cavity for PIP2, a common modulator for ion channels whose binding cavity is located within the intracellular C-terminal domain [11], TRPM8 comprises a second more specific pocket placed within the intracellular sensor module composed by the S1–S4 segments and ligands interacting with this can elicit both agonist and antagonist activity [12]. Mutational analyses allowed a precise characterization of the arrangement of this pocket and the key interacting residues [13]. This binding site immediately appeared to be a potentially druggable cavity, especially because its non-conserved residues should permit the rational design of reasonably selective ligands [14].

On these grounds and in parallel to the finding of novel therapeutic applications, the interest for TRPM8 ligands is progressively increased and consequently several agonists, antagonists and modulators have been reported in the literature coming from both academic and industrial community [15]. Similar to what happened for other TRP channels, the lack of experimentally resolved TRPM8 structure strongly limited the role of structure-based in silico approaches and however, ligand-based approaches were rarely applied to the identification of TRPM8 ligands. For many years, the only structure available for TRPM8 was a homology model developed by some of us and based on a fragmental strategy, which combined different templates [16]. While considering its significant inaccuracies, this model showed an encouraging predictive power even when applied for virtual screening campaigns as seen for the identification of a novel set of naphthyl ligands [17].

Recently, the tetrameric structure of TRPM8 from *Ficedula albicollis* was resolved by cryo-electron microscopy in its unbound state as well as in complex with WS-12 (a menthol analog) or icilin (plus in both cases PIP2 and Ca^++^). [18,19] While not corresponding to the human protein, the high homology between hTRPM8 and faTRPM8 renders these resolved structures truly reliable templates to generate a complete and accurate homology model for the human TRPM8 channel in its tetrameric structure. Homology models based on faTRPM8 were already proposed in the literature and found fruitful applications in docking simulations as recently reported for the identification of a set of tryptophan-based antagonists. [20] Nevertheless, no study described until now the application of these improved hTRPM8 homology models in virtual screening campaigns.

Hence, we have undertaken a comprehensive study aimed at assessing the reliability of the here generated hTRPM8 homology model in its tetrameric structure as well as at developing optimized computational strategies for virtual screening (VS) simulations by performing extended benchmarking analyses, based on a progressive ensemble docking strategy, which involved different hTRPM8 conformations, docking programs, scoring functions and consensus approaches.

In detail, the hTRPM8 tetrameric model was generated based on the unbound faTRPM8 structure and underwent an initial 1.0 μs MD simulation, which had two primary objectives: first, to allow a reasonable equilibration of the entire tetrameric structure and then to explore the dynamic behavior of the four monomers. Indeed, and although simulated in its unbound state, one may imagine that the TRPM8 tetrameric structure retains a certain degree of flexibility and the four interacting monomers influence each other by inducing structural fluctuations that can also influence the fine architecture of the four binding sites within the sensor modules.

Hence, besides monitoring the dynamic equilibration of the TRPM8 tetramer, the MD run was also utilized as a valuable source of multiple receptor conformations to be used in the following docking simulations [21]. In detail, the frames were selected based on their similarity with the resolved structures in complex with icilin and WS-12 [19]. Notably, the chosen TRPM8 tetrameric structures were utilized by repeating the docking calculations on all the four monomers. As mentioned above, docking simulations involved different pieces of software, and indeed, another relevant objective of this study was focused on the evaluation of the LiGen™ tool [22] in comparison with other well-known docking programs, such as GOLD and PLANTS. 

After a preliminary indirect re-docking study performed to select the most interesting frames as well as to calibrate the settings of the various docking programs, virtual screening campaigns involved the same database utilized in our previous study, which includes 53 known TRPM8 antagonists combined with 4947 true inactive molecules as derived from high-throughput screening. [17] For each selected TRPM8 structure, the four corresponding monomers underwent docking simulations and the computed complexes were rescored by ReScore+ [23] and then utilized to develop consensus equations based on the recently proposed EFO method [24]. As detailed in the results, highly effective consensus equations were generated when combining the docking results as computed for the four monomers of the same TRPM8 tetramer. Such a result confirms that the binding cavities of the four monomers undergo limited but not negligible conformational changes, which render them representative of the different states that TRPM8 can assume during recognition processes. The here reported approach can thus be seen as a general strategy that can be applied to account for the dynamic behavior of oligomeric protein targets with multiple identical binding sites by simultaneously considering all single monomers.

## 2. Results

### 2.1. hTRPM8 Homology Model

As detailed in the methods, a homology model of human TRPM8 (hTRPM8) was generated using the recently published Ficedula albicollis TRPM8 (faTRPM8) structure as the template [18]. Such a resolved structure shows significant differences concerning TRPV1, TRPV2, and previously reported TRPM8 models, and assigns the location of several residues involved in modulator binding site. Focusing on the region formed by the TM helices S1−S6 and the TRP box (residues 723−1013), the hTRPM8 monomer was modeled by using the Prime software [25]. 

**Figure 1:**
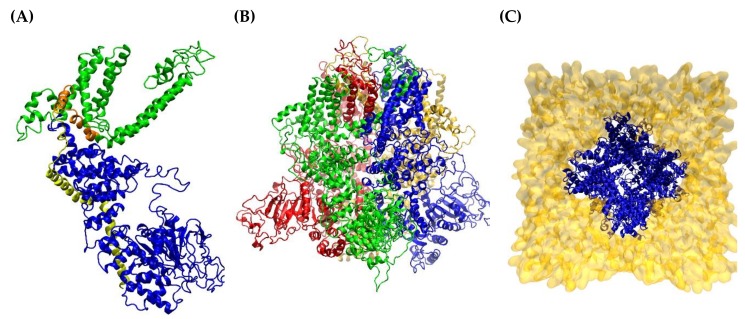
Structure of the generated hTRPM8 homology model in its monomeric (**A**), colored by segments: blue = N-terminus, green = transmembrane helices, yellow = C-terminus, orange = TRP domain) and tetrameric (**B**), colored by monomer, lateral view, green, blue, yellow and red respectively and (**C**), embedded in the phospholipid bilayer, top view) forms. Apart from water molecules, Figure (**C**) represents the system that underwent the MD run.

Figure 1 shows the ribbon structure of the generated hTRPM8 homology model as seen parallel to the membrane in its monomeric (1A) and tetrameric (1B) form. In detail, the monomer reveals the well-known arrangement of the six transmembrane bundle, which identifies a sensore module composed of the first TM segments (S1−S4) that harbor the here explored binding pocket and a pore module that comprises the last two TM helices (S5−S6). The two modules are connected by an S4−S5 loop, which shows an alpha-helix structure and the conformation of which modulates the arrangement of the pore module and consequently the pore opening. Similarly to other ion-channels, the long S5−S6 loop shows a complex arrangement with a portion that assumes an α motif and appears to be reinserted within the transmembrane region. Both terminal domains are inserted in the cytosol and show mixed α/β secondary motifs. Remarkably, the C-terminus accommodates the binding site of PIP2 and more importantly approaches the sensore module and is involved in the overall definition of its binding cavity. The tetramer appears to be stabilized by the interactions between the helices forming the pore and the N-terminal domains which show a tightly fitted arrangement that has a clear role in tetramer stabilization. In contrast, the extracellular portion seems to have a limited relevance and only the disordered portion of the S5−S6 loop significantly protrudes from the membrane region. The central pore defined by the tetramer is appreciable when viewing the tetrameric assembly embedded in the phospholipidic bilayer for the MD simulation from the extracellular side as displayed in Figure 1C.

It is worth noting that the modeled hTRPM8 shows a high similarity with the faTRPM8 template, and this similarity increases when focusing on the binding region delimited by the sensore module domain. Interestingly, the modeled binding site shows a satisfactory similarity not only with the used template (6BPQ = 2.14 Å) but also with the recently deposited TRPM8 structures in complex with icilin and WS-12 [19]. In detail, an RMSD analysis was carried out by comparing S1−S4 and TRP domains and revealed that the greatest similarity is shown with the resolved TRPM8 in complex with icilin (6NR3 = 1.79 Å) and however all comparisons are characterized by rmsd values lower than 2.5 Å (6NR2 = 2.28 Å and 6NR4 = 2.36 Å) to confirm a significant similarity between the generated homology model and the available resolved TRPM8 structures.

### 2.2. MD Simulation

**Figure 2:**
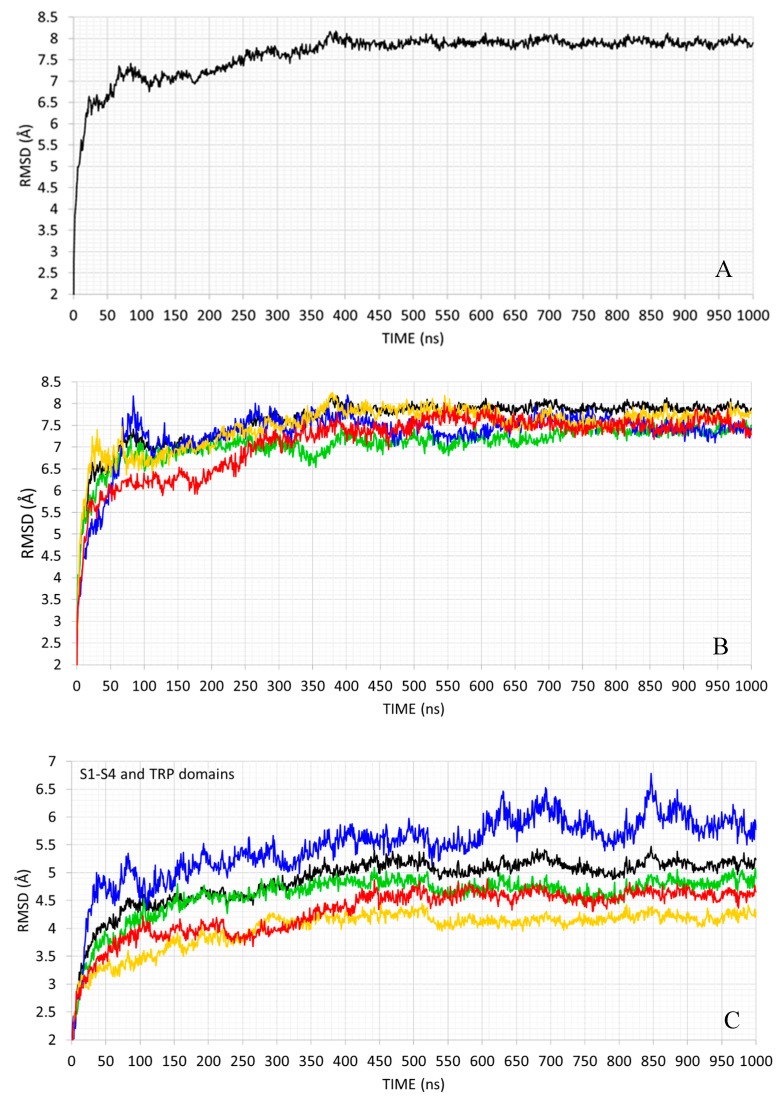
Atomic fluctuations (RMSD in Å) as computed by considering the backbone heavy atoms of the entire tetramer (**A**), the single monomers (**B**) and the residues belonging to the simulated binding pocket ((**C**), green, blue, yellow and red for monomer A, B, C, and D, respectively).

MD run had two primary objectives: (1) to assess the stability of the modeled hTRPM8 tetramer and (2) to explore its conformational space to select protein conformations particularly suitable for the following docking simulations. Concerning the first aim, protein stability was investigated by calculating the backbone rmsd profiles. Figure 2A reports the rmsd values for the entire tetramer and reveals how the initial 350 ns are required for tetramer equilibration. Then, a reliable structural stability was reached, with RMSD values fluctuating within the range of 1.0 Å during the following simulation time. When focusing attention on the single monomers, Figure 2B shows that they have very comparable behaviors, suggesting that they equally participate in tetramer stabilization. Table 1 decomposes the rmsd profiles in the structural segments as computed for each chain and reveals that the largest fluctuations are observed in the terminal domains, while the transmembrane portion (S1−S6 helices) and the TRP domain show a reduced mobility. There are no marked differences between chains even though one may observe that Chain A shows on average the lowest mobility while chain B appears to be the most mobile one. This similar behavior exhibited by the four monomers is also explainable by considering that the simulated protein is a symmetric homotetramer and the performed MD simulation is long enough to reach and retain this overall symmetry.

Greater differences between monomers are observed when focusing attention on the binding cavity as defined by S1−S4 (residues 736−757, 769−788, 797−816, 824−851) and TRP (residues from 992 to 1008) regions. Indeed, Figure 2C shows that the chains A and C reach a satisfactory structural stability after about 300 nanoseconds (ns) of simulation (more evident for the chain C), which is then maintained for the whole duration of the calculation. The chain D reaches a stable conformation around at 450 ns, while the chain B is characterized by significant fluctuations, which never allow a reliable structural stability to be reached. The local differences observed between monomers can be justified by considering that the protein is simulated within an anisotropic medium (the POPC-based membrane model) and so even limited differences in the arrangement of the surrounding phospholipid molecules can induce local changes in the protein conformations. Although randomly induced, these local conformational changes allow a more extended sampling of the arrangement of the binding cavity. Moreover, the different behavior of the simulated binding sites appears to be suggestive of a mutual influence between monomers that affects the fine architecture of the four binding sites. This justifies the involvement of all four monomers of the selected frames in the following docking simulations.

**Figure 3:**
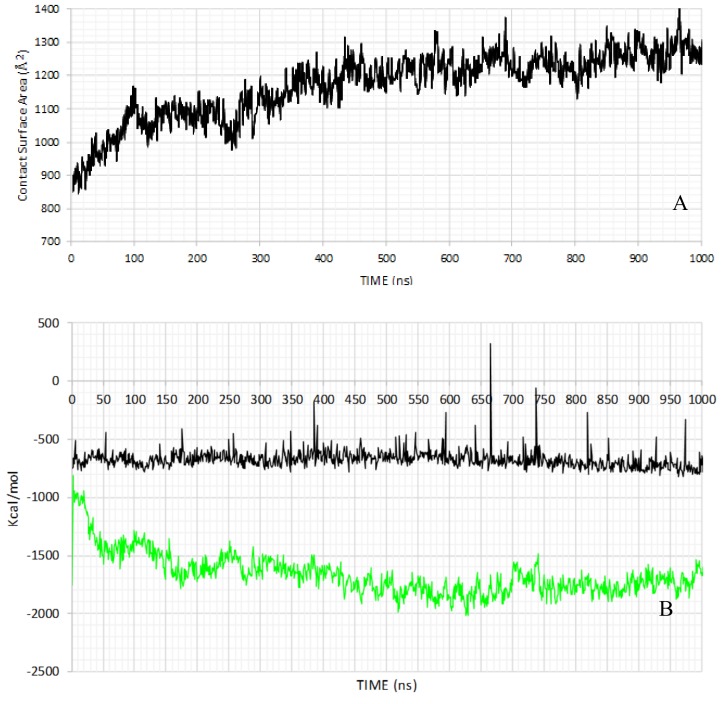
Dynamic profile as monitored during the MD run for the intermonomeric contact surface (**A**) and the electrostatic and Lennard-Jones interaction energies as computed between the monomer A and the remaining trimer ((**B**), black and green lines for electrostatic and Lennard-Jones energy values, respectively).

To better investigate the stability of the simulated TRPM8 tetramer, Figure 3A reports the dynamic profile of the inter-monomeric contact surface, which is computed as detailed in the methods section. The monitored inter-monomeric contact surface mostly involves the very large N-terminal domain, while S1, S4 and the S5−S6 loop appear to be the most relevant segments concerning the transmembrane bundle. The computed contact surface reveals a profile that brings to mind that of the rmsd values. Indeed, the surface values increase in the first 350−400 ns to remain roughly constant in the remaining part of the simulation. Such a similarity suggests that the conformational fluctuations seen in the first part of the MD run are reasonably driven by the stabilization of the quaternary structure, which involves a progressive increase of the inter-monomeric interactions. To analyze the kinds of intermolecular interactions that play a crucial role in the observed tetramer stabilization, Figure 3 reports the dynamic profiles for the Van der Waals interactions as computed by the Lennard−Jones equation using the CHARMM force-field and the electrostatic energy by applying a distance-dependent dielectric function. For simplicity, the analysis was conducted considering only one representative monomer (chain A). Figure 3B indicates that the quaternary stabilization is primarily due to the strengthening of the ionic interactions that indeed show a marked increase in the first 350 ns, while Van der Waals interactions display a roughly constant behavior throughout the MD run.

### 2.3. Frame Selection

Although the available resolved structures would allow the generation of reliable hTRPM8 complexes to be simulated, the here performed MD simulation involved the unbound hTRPM8 structure to explore its conformational space in a completely flexible and unbiased manner as well as to investigate if the collected frames were able to reproduce the conformations of the bound resolved structures at least, concerning their binding cavity. Hence, the MD results were analyzed in order to extract a set of representative structures to be used in the following docking simulations [26]. This analysis involved only the frames derived from the second part of the simulation, after 350 ns when the complex appears to be suitably stabilized.

**Figure 4:**
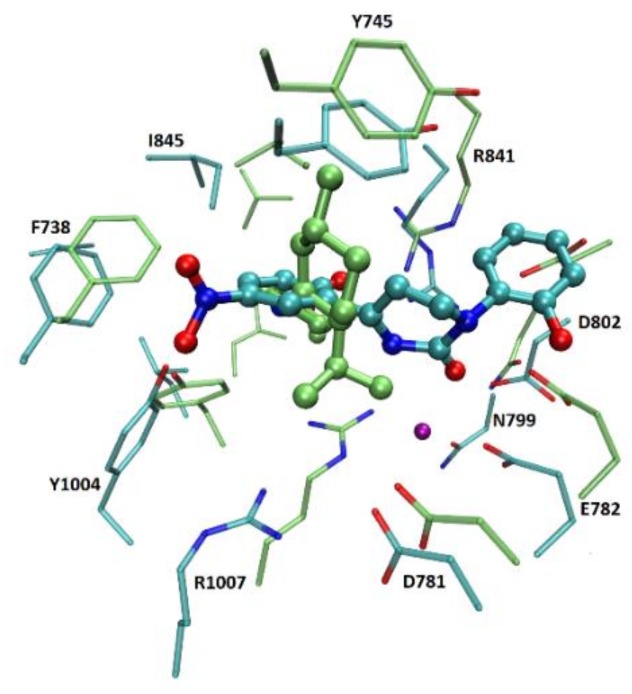
Superposition between 6NR2 (green lines and stick) and 6NR3 (cyan lines and stick) active site residues. The picture shows the differences in the binding site, mainly related to Glu782, Ans799, Asp802, those that interact with Calcium atom (magenta sphere), and Arg1007.

The selection was based on the RMSD between the frames taken from the MD simulation and the recently crystallized structures of TRPM8 (i.e., 6NR2, 6NR3, and 6NR4 [1]). The comparison was focused on 6NR2 and 6NR3, as 6NR4 is a low occupancy structure in which the precise pose of the ligand is unresolved. Indeed and as depicted in Figure 4, 6NR2 and 6NR3 show a superimposable overall structure but their binding sites reveal not negligible differences mostly focused on the residues which only in 6NR3 contact the calcium ion. In detail, we measured the RMSD of the whole backbone, the transmembrane helix S1−S2−S3−S4 and the binding site residues (Y745, I746, L778, E782, Q785, N799, D802, F838, R841, H844, E1003, Y1004, and F1012). Similarly to what was observed for the RMSD profiles in the previous analyses, the RMSD of the backbone and the transmembrane helix do not significantly change during the simulation, while the RMSD values of the binding site of the four chains reveal significant fluctuations that allow a reliable selection of the seemingly most representative protein conformations (Table 2).

Considering the binding site backbone, we obtained more than one frame with an RMSD close to 2 Å, while considering all the heavy atoms of the binding site residues the best RMSD values appear to be between 3 and 4 Å. As shown in Figure 4, the higher RMSD values, as computed by considering the binding site side chains emphasize the relevant differences between the two considered structures and justify the comparison performed here with both 6NR2 and 6NR3. There are no marked differences between the lowest RMSD values with 6NR2 and 6NR3 meaning that the MD run can be conveniently utilized to extract conformations in which the binding site compares well with those in complex with both icilin and WS-12.

In order to select the frames to be utilized in the following virtual screening campaigns, an indirect re-docking study was performed by docking icilin and WS-12 in the previously selected frames and comparing the so computed poses with the corresponding poses in the resolved reference structures (6NR2 and 6NR3). Table 3 compile the computed rmsd values as obtained using PLANTS, GOLD, LiGen™, and Glide on the non-minimized (3A) or minimized (3B) protein structures.

The reported rmsd values allow for some relevant considerations. First, and similarly to what was observed in previous studies, [27,28] the non-minimized structures afford, on average, better results than the minimized ones. However, a minimized frame (990) shows quite low rmsd values and thus was selected for the following virtual screening campaigns. Second, WS-12 reveals better poses than icilin and this difference might be due to the rotation of 180° of the ligand, compared to the crystal structure. When considering the experimental uncertainty in the Ying Yin’s work [1], one may hypothesize that icilin can bind to TRPM8 with two symmetrical binding modes: the one present in the crystal structure where the para nitrophenyl group of Icilin interacts with the Tyr1004 and the phenol with Phe838 and Tyr745 as well as a second binding mode in which the para-nitro phenyl moiety elicits hydrogen bond and π–π stacking interactions respectively with Tyr745 and Phe838, while the phenol group is oriented towards the Tyr1004. Third and on average in all simulations, LiGen generates the best results, GOLD, and Glide affords comparable intermediate rmsd values and PLANTS appears to be the worst-performing software. On these grounds and considering the frames which obtained the lowest rmsd values (listed in bold), virtual screening campaigns involved the non minimized 652 and 1049 frames as well as the minimized 990 frame.

**Figure 5:**
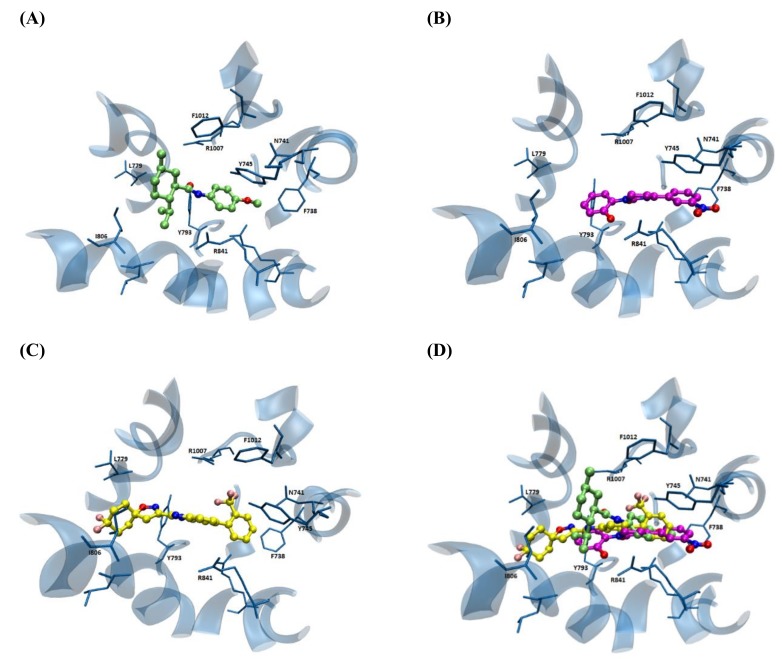
Main interactions stabilizing the putative complexes between the hTRPM8 monomer A and WS-12 (**A**), icilin (**B**) and the benzimidazole-containing antagonist (**C**). Figure (**D**) compares the three computed poses.

In order to catch a glimpse of the simulated binding sites, Figure 5 reports the computed complex involving the binding pocket of the monomer A of the frame 1049 (chosen due to its remarkable performances in the following VS campaigns, see below) and some selected ligands (i.e., the agonist WS-12 and icilin and a benzimidazole-containing antagonist). In detail, Figure 5A shows the putative complex for WS-12, which appears to be mostly stabilized by the rich set of contacts elicited by the phenyl ring involving both π−π stacking (with Phe738 and Tyr745) and charge transfer interactions (with Arg841 and Arg850). Moreover, the para methoxy group elicits a reinforced H-bond with Arg850 plus hydrophobic contacts with Ile845. The substituted cyclohexane ring is involved in a set of apolar interactions with surrounding alkyl side chains such as Leu774, Val775, Leu778, Leu779, and Ile806. Finally, the amide linker is not involved in direct contact even though it can stabilize water-mediated H-bonds with Glu762 and, to a minor extent, Arg841 and Arg1007.

Figure 5B reports the computed complex for icilin and reveals a more marked relevance of the polar interactions compared to the previous one. In detail, the para-nitro phenyl moiety is involved in π−π stacking interactions with Phe738 and Tyr745, which can also stabilize weak H-bonds with the nitro group, while the ortho phenol ring contacts Tyr793 and the hydroxy function elicits clear H-bonds with Asp802 and Arg841. Finally, the 3,6-dihydro pyrimidine-2-one ring stabilizes H-bonds with Arg850 and to a minor extent with Lys792.

Figure 5C shows the computed complex for the benzimidazole-containing antagonist chosen since it is the most active compound included in the dataset used in the following VS campaigns. This reveals a combination of polar and hydrophobic contacts even though the latter appears to have a preeminent role. In detail, the trifluorophenyl ring stabilizes a rich set of both π−π stacking interactions with Phe738 and Tyr745 and charge-transfer interactions with Arg841 and Arg850. Again, the trifuolomethyl group is seen to elicit a halogen bond with Tyr745. The central benzimidazole ring is involved in charge-transfer interactions with Arg841 and Arg1009, while the spiro-isoxazoline system mostly elicits apolar contacts involving Val783, Leu789, Leu804, and Ile806.

Figure 5D compares the obtained poses for the three considered ligands and reveals that the two agonists roughly share the same occupied region even though icilin is stabilized by a richer set of polar contacts compared to WS-12 as described above. In contrast, the simulated antagonist can reach a hydrophobic subpocket not involved with the agonists, where it arranges its spiro-isoxazoline moiety. 

**Figure 6:**
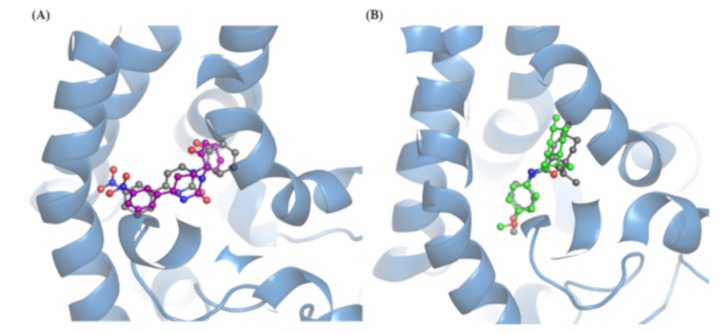
A schematic comparison between docking and crystal structure poses for icilin (**A**) and WS-12 (**B**). The proteins, extracted from MD, are reported in blue cartoon; the co-crystallized and docked ligands are reported in grey and colored stick respectively.

Finally, Figure 6 compares the best docking pose with the crystal structures for both icilin (6A) and WS-12 (6B) and reveal an encouraging superimposition between them. On one hand, this satisfactory result which can be seen as a sort of indirect re-docking confirms the reliability of here developed docking strategies. On the other hand, this underlines the capacity to extract binding site conformations amenable for docking simulations from the here performed MD run.

### 2.4. Virtual Screening Campaigns

By considering the previously discussed results, virtual screening (VS) simulations involved the three selected frames and were performed using PLANTS, GOLD, and LiGen™ programs. As mentioned in the Introduction, these VS analyses involved all the four monomers included in the selected structures and the complexes computed by each program were then rescored by ReScore+. This means that each docking simulation was parameterized by the primary scoring function(s) directly computed by the used program plus a set of representative scores computed by ReScore+. For each docking program, the so obtained results were then utilized by a progressive ensemble docking approach which can be subdivided in three steps. The first step involved the scores computed for each single monomer and single frame. The second step combined the results obtained for the four monomers of each single frame, while the last step considered simultaneously the docking results obtained for all frames and all monomers. In all steps, consensus equations were generated by linearly combining up to four scoring functions using the EFO approach [24]. 

#### 2.4.1. Virtual Screening Results for Each Monomer of Each Frame

Table 4 compares the obtained best EF1% values, considering separately the docking results of each frame and each monomer, plus the corresponding mean values as computed per monomer, per frame, and docking program. The reported EF 1% values were obtained by considering the best performing single primary score as well as the best consensus equations based on the EFO approach, which also comprises the results from the rescoring analyses. The reported results allow for some relevant considerations. The first general consideration involves the remarkably beneficial effect exerted by the rescoring calculations and linear combinations of diverse docking scores that appear to be noticeable in all VS campaigns regardless of the docking program. On average, the generation of consensus models elicits a three-fold improvement of the EF 1% values with PLANTS, which shows the largest seven-fold enhancement. As observed in previous studies [25], there is a general relation between the one-score performances and those obtained by rescoring and consensus approaches and this finding confirms that these post-docking calculations can enhance the general performances but cannot completely escape the drawbacks of the used docking programs. 

When comparing the performances for the utilized pieces of docking software, one may note that LiGen™ affords the highest EF 1% values both when considering the best performing primary scores and when generating consensus models. PLANTS affords intermediate performances, which however greatly benefit from rescoring and consensus approaches (as mentioned above), while GOLD produces modest results even when developing consensus equations. Interestingly, the satisfactory results yielded by LiGen™ are in agreement with those previously obtained by the initial indirect re-docking, while the performances of PLANTS and GOLD appear to be in contrast with what was observed in the initial docking studies. This may suggest that PLANTS docking scores are more effective in ranking the simulated ligands rather than in generating the correct poses. 

When comparing the performances obtained by the three selected frames, the computed overall mean values reveal that the frame 1049 performs markedly better than the other two frames, which in turn shows comparable results. These differences are largely due to the different performances by LiGen™ and PLANTS, while GOLD provides poor results with all frames. Even though the performances of the single monomers do not show similar trends in the three selected frames, the monomers A and B provide the best and the worst EF 1% values in all frames, respectively. Remarkably, the binding site of the monomer A was able to rapidly reach an equilibrium state during the MD run (as seen in Figure 3), while the monomer B never reached an equilibrium state showing large fluctuations during the entire simulation. This suggests that the capacity to promptly reach a reliable conformational stability as exhibited by the monomer A results in binding sites more often suitable to accommodate the ligands, while the prolonged instability of the monomer B reduces its capacity to assume suitable arrangements.

The analysis of the consensus models based on the LiGen™ program and generated by the EFO approach for all frames (Equations not shown) confirms the remarkable performances of the LiGen™ scores and reveals the beneficial role of both MLPInS to encode for hydrophobic contacts [29] and the number of contacts, the recently proposed scores to parameterize the ligand fitting in terms of stabilized contacts [26]. Similar results are provided by the PLANTS consensus models in which the primary scores are often combined with MLPInS functions and the number of contacts. As an example of these consensus models, the linear equations generated using LiGen™ with the subunit A of frame 990 is worth mentioning due to its outstanding EF 1% value equal to 65.25 corresponding to 35 active ligands among the 50 top-ranked molecules. 

#### 2.4.2. Virtual Screening Results Combining the Four Monomers of a Single Frame

Table 5 reports the best consensus equations as developed when combining the docking scores computed for the four monomers of each frame. The equations have to include docking scores coming from at least two different monomers to avoid equations identical to those already considered in Table 4. As a preamble, it should be noted that the docking results generated by GOLD did not allow the generation of satisfactory models even when combining the four monomers of a frame and thus the corresponding models were not included in Table 3 for simplicity. Such an unsatisfactory result parallels the modest performances obtained by GOLD when considering separately each monomer and further confirms that consensus and rescoring methods, however sophisticated, cannot upset the initial docking performances.

The first consideration involves the comparison between the best performances reported in Table 4 and those reached considering the monomers separately. Apart from the outstanding performance exhibited by LiGen™ with the monomer A of the frame 990 (see Table 3), in all the other five cases the combination of the four monomers enhances the performances compared to those reported in Table 3. The enhancement is greater when using the PLANTS docking results compared to LiGen™, and this can be easily explainable by considering that LiGen™ yields very remarkable EF 1% values even by considering single monomers and thus its performances can be further improved with difficulty. 

The analysis of the involved monomers in these multi-monomer consensus equations reveals that monomer A is the only one that is included in all reported equations. In detail, about half included docking scores (10 out of 21) refers to monomer A, while the other three monomers show a lower and comparable role in the consensus equations. This result is in agreement with what was observed when considering single monomers separately and further confirms that the marked stability shown by monomer A during the MD simulation implies that it can assume conformations more suitable for ligand binding. Nevertheless, the beneficial effect elicited by the combination of more than one monomer indicates that also the other monomers, while having less suitably arranged binding sites, can contribute to overall ligand binding. As seen above and along with the primary scores, the included scoring functions confirms the reliability of MLPInS functions and the number of contacts, which represent about half included docking scores (10 out of 21). In agreement with what was observed for the single monomers, the comparison of the performances for the three selected frames reveals that the overall best results are provided by frame 1049, followed by 990, and lastly 562, while the best model is also here provided by frame 990 using LiGen™. 

#### 2.4.3. Virtual Screening Results Combining the Four Monomers of all Selected Frames

Table 5 also includes the best consensus models developed by combining the docking scores coming from all the performed VS campaigns, i.e., the four monomers of the three selected frames. Intending to develop solid and effective models including as few as possible variables, Table 5 reports the best equations with 2, 3, and 4 docking scores. They were selected provided that they include the scores from at least two different frames to avoid results identical to those previously described. For completeness, Table 4 also includes the EF 1% values of the best performing single score as derived using PLANTS and LiGen™.

As seen in the previous analyses, the first consideration concerns the better performances reached by the LiGen™ docking results when comparing the equations including 2, 3 and 4 variables. Interestingly, the combination of more than one docking score has effects comparable by both programs, which progressively decrease with the number of included variables. In detail, the inclusion of two variables induces in both cases an EF 1% increase greater than 15; adding a third score leads to an EF 1% enhancement around 10, while equations with four variables reveal a similar and marginal enhancement (around 2.0). These results suggest that the effect of a linear combination of docking scores is here reaching a plateau condition with four different variables, thus justifying the choice of avoiding more complex consensus equations that would have very limited effects with the overfitting risk. As a matter of fact, it should be noted that LiGen™ allowed the generation of the best performing consensus model thus emphasizing the remarkable performances of this docking tool as well as the efficacy of ensemble docking strategies based on all monomers and more than one representative frame.

With regards to the involved monomers, the reported equations reveal that the monomers A and D show a marked and comparable relevance, while the monomer B is the least utilized. These results are in line with what was observed in the previous sections and can be easily interpreted in terms of different stabilit of the four monomers. Concerning the involved frames, the comparison between the LiGen™ and PLANTS results reveal significant differences. Indeed, the PLANTS equations include docking scores coming from the frames 562 and 1049 (both frames with five occurrences), while the LiGen™ models also comprise scores from frame 990, which even shows the highest frequency. These different results might indicate that PLANTS prefers non-minimized protein structures, while LiGen™ is also able to exploit minimized protein structures thus showing a greater versatility concerning the structural requirements of the protein target. 

## 3. Discussion

To the best of our knowledge, the here reported results represent the first case in which the recently resolved TRPM8 structures were utilized and proved successful in performing virtual screening campaigns. Thus, the present study was primarily focused on the development of purposely targeted computational strategies with a view to optimizing the predictive performances of the VS analyses involving an extensively simulated tetrameric hTRPM8 model based on the resolved faTRPM8 structure in its unbound state. 

The MRC docking approach has been useful in identifying new hits and leads in several biochemical pathways and targets. This method aims to reproduce a conformational selection mechanism, in which specific protein conformations are recognized by ligand(s) to form a thermodynamically favored protein-ligand complex [30]. Moreover, MD simulation successfully achieved the two major objectives for which it was performed. Firstly, it allowed a satisfactory equilibration of the modeled tetramer indicating that, after the first 350 ns, the system remains substantially stable during the time. More detailed analyses revealed that such an equilibration process is mostly driven by a stabilization of the quaternary structure which involves an increase of the inter-monomeric interactions by a strengthening of the ionic contacts. The conformational profile of the single monomers appears to be relatively stable although the region defining the considered binding pocket reveals a certain degree of structural variability even in the second part of the MD run with clear differences between the monomers. Secondly, the structural flexibility of the binding pocket allowed a fruitful exploration of its conformational space, thus extracting some representative frames to be used in the following docking simulations.

Concerning docking simulations, the obtained results allow for some meaningful considerations. First, the comparison of the utilized docking programs clearly emphasizes the superior performances offered by LiGen™ both in generating consistent poses as seen in the preliminary docking simulations and ranking and discriminating active and inactive molecules as appreciated in VS analyses. PLANTS provides intermediate performances that however greatly benefit from rescoring and consensus approach. Finally, GOLD yields modest results in VS campaigns, while proving successful in calculating reliable poses as evidenced in the initial docking calculations. 

Second, rescoring and consensus methods elicit markedly beneficial roles in almost all performed docking analyses. However, and in line with what was noted in previous studies, there is a clear relationship between the performances reached by the primary scores alone and those reachable by the following rescoring and consensus calculations. This means that these post-docking simulations are heavily dependent on the reliability of the computed docking results and cannot go beyond the limitations imposed by the used docking software. Overall, this study affords a compelling confirmation of the noticeable potentials of the consensus approaches based on linear combinations of docking scores as implemented in the EFO algorithm.

Third, all performed docking simulations benefit from simultaneously considering, in the consensus approaches, the docking scores from the four monomers of a given frame. The scoring functions of the various monomers appear in the generated consensus models with different frequencies which can be related to the structural stability of the involved monomers; however, all monomers seem to play a role in the equations thus suggesting that even the seemingly less-stable monomers can have a (limited but not negligible) role in binding processes. Overall, these results emphasize the cross-talk processes by which monomers mutually influence each other by modulating the fine architecture of their binding pockets. From a conformational standpoint, such a mutual influence is reflected in the different stability evidenced by the binding pockets during the MD run (see Figure 2C), while its effects on ligand binding can be taken into consideration by performing and combining docking simulations on all four monomers. Also combining various frames extracted by the MD simulation has a beneficial effect and this is in line with the numerous studies based on the so-called ensemble docking approaches [31]. 

However, the comparison of the EF 1% enhancements reported in Table 5 highlights that combining the four monomers elicits an effect that is slightly lower than that obtained by also combining all selected frames. This finding means that most of the beneficial effect exerted by the here explored consensus strategies is due to the monomer combination while taking into account different frames affords more limited effects. This result depends on the criteria adopted for the frame, selection and different effects might be obtained by a more exhaustive sampling of the performed MD simulation. Here, the frame selection was based on a similarity criterion aimed at minimizing the computational cost, while considering a reasonably suitable binding pocket and in this context, the beneficial role of the monomer combination appears very remarkable. Such a result can also have a very general relevance and suggests that such a computational strategy can be always adopted by structure-based studies involving multiple conformation targets with more than one identical binding site. 

## 4. Materials and Methods 

### 4.1. Monomer Generation

The primary sequence of the human transient receptor potential melastatin 8 (hTRPM8) was retrieved from the UniProt database (UniProt code: Q7Z2W7), and the sequence was aligned to *Ficedula albicollis* TRPM8 (faTRPM8; UniProt code: U3JD03, PDB ID: 6BPQ [18]) using MUSCLE v.3.8 [32]. The three-dimensional atomic models of the homology TRPM8 monomer model was then generated by using the energy-based model implemented in Prime [24], by using the chain A of faTRPM8 as the template. The obtained homology model was submitted to the Schrödinger Protein Preparation [33] tool to optimize the 3D structure from a chemical and conformational point of view. In detail, hydroxyl, thiol and amide groups, as well as the His imidazole rings, were rearranged to optimize the hydrogen-bonding network. Again, the His, Asp, Glu, Arg, and Lys ionization and tautomeric states have been modified to be compatible with the physiological pH equal to 7.4. The last step of the optimization workflow consists of a structure restrained minimization (OPLS2005 force field) [34] that was stopped when the rmsd value of the heavy atoms converged to 0.30 Å. Ramachandran structure analysis was performed using PROCHECK v.3.5 [35], and the so prepared hTRPM8 homology model showed 78.5% residues in the allowed regions, 19.8% residues in favorably allowed regions, and only ~1.87% in the disallowed regions, meaning that most of the amino acids (98.3%) fall in allowed phi/psi regions. 

### 4.2. Tetramer Assembly and MD Simulation

The TRPM8 tetramer was then assembled by aligning the four monomers on the resolved faTRPM8 structure (PDB ID: 6BPQ [18]) and superimposing the backbone heavy atoms of all segments comprising extra-, intra-cellular regions, and the transmembrane elements. To obtain a reliable model, the TRPM8 receptor was embedded into a palmitoyl phosphatidylcholine bilayer, by using the OPM server [36]. Next, the Prime module was used to optimize the loops and to minimize the structure, considering the membrane explicitly. To assess the structural stability of the protein-membrane system and to explore its conformational space, 1000 ns (1.0 µs) of molecular dynamics simulations were run. MDs calculation was carried out by using Desmond Multisim protocol [37]. The whole system was solvated in an orthorhombic box with a buffer of 10 Å TIP3 (transferable intermolecular potential three-point) water molecules and Na+ counter ions were added to neutralize the system net charge. Overall, the so generated system included 532666 atoms, of which 121490 water molecules, 720 POPC molecules composing the membrane and 16 counter ions molecules. In the early stage, the Multisim method allowed the structure to equilibrate and relax, simulating a mature system. The calculation was run under constant pressure of 1 atm and a temperature of 310 K, thermostated and barostated according to the Martyna–Tobias–Klein method, with a coupling constant of 0.5 (2.0) ps for the thermostat (barostat). The whole hydrogen positions were constrained by the M-SHAKE algorithm, allowing a time step of 2 fs. The long-range electrostatics were computed at every time step by the PME (Particle Mesh Ewald) method with a cut-off radius of 10 Å. The here analyzed contact surface is computed by using the following equation: σ(t) = (S_monomer_(t) + S_trimer_(t) – S_tetramer_(t))/2, where S_monomer_(t), S_trimer_(t) and S_tetramer_(t) refer to the solvent-accessible surface area as computed for monomer A (taken as reference), remaining trimer and entire TRPM8 tetramer, at time t, respectively. These surface values were computed by the VEGA suite of programs [38] extending each van der Waals radius of a user-defined probe radius equal to 1.4 Å.

### 4.3. Virtual Screening Analyses

Docking simulations involved the same dataset already utilized in a previous study, which is composed of 5300 molecules, 53 of which are known binders and 5247 are experimentally proven as known-binders. Each ligand was prepared by considering the predominant form at physiological pH as described in [17]. Additionally, all collected molecules were minimized by semi-empirical methods using the PM6 Hamiltonian as implemented in MOPAC [39]. The reliability of the prepared dataset was further confirmed by applying the EFO method (see below) on a set of well-known physicochemical and stereo-electronic descriptors as computed by VEGAZZ and MOPAC. The best predictive model generated by including two variables reaches an EF 1% around 5, a result which is far below the performances obtained by docking scores and which suggests that the dataset should not include marked biases (between active and inactive molecules) as to affect the here presented results. The preliminary docking analyses and the virtual screening campaigns were performed using three programs: PLANTS [40], GOLD [41], Glide [42] and LiGen™ [22]. All docking simulations were focused on a binding pocket defined by a 10 Å radius sphere around the center of mass described by the residues Tyr745, Asn799, Asp802, and Tyr1004, chosen because of their well-known role in ligand recognition [18]. Docking simulations by using PLANTS and GOLD were performed using the ChemPLP scoring function, the speed1, and the virtual screening accuracy method, respectively. Glide simulations were performed using the standard precision (SP) search algorithms of Glide software, considering the ligand structural flexibility. Default Glide scoring function (GScore) was applied for poses ranking. The geometrical docking procedure implemented in LiGen™, which follows a specific workflow to compute three docking scores, was used for the docking simulations. First, the Pacman Score (PS) estimates a geometric fitting score to evaluate the interaction between a ligand conformation and the pocket, basing on shape and volume information; then the Chemical Score (CS), representing the ligand binding energy, is calculated by using an in-house developed scoring function [22]. Lastly, a minimization algorithm that treats the docket ligand as a rigid body inside the binding site, called the Optimized Chemical Score (Csopt) is evaluated. Furthermore, all computed poses produced during the virtual screening campaigns were rescored using Rescore+. The primary scoring functions plus the scores computed by rescoring were utilized to generate consensus linear equations using the Enrichment Factor Optimization (EFO) method [24], as implemented in the VegaZZ suite of programs. Such an approach develops consensus models by linearly combining the input docking scores by an optimization procedure that maximizes the corresponding EF 1% value [25]. To validate the generated consensus models the input dataset is randomly subdivided into training (70%) and test (30%) sets and to minimize the randomness this task is repeated five times. 

### 4.4. Hardware Details

Docking and Molecular Dynamics calculations were run on a proprietary Quad-Socket Server equipped with 4x Intel® Xeon® Platinum 8176 Processor 28 Cores (56 Threads), 24x DDR4-2666 MHz 32GB and 2x GPU NVIDIA Quadro GV100 32GB.

## Figures and Tables

**Table 1:** Atomic fluctuations (RMSD in Å) as computed by considering the backbone heavy atoms and by averaging each structural segment for each chain.

	S1−S6	TRP	N-TER	C-TER	Average
**Chain A**	2.56	1.60	3.65	2.55	2.59
**Chain B**	2.89	2.12	4.24	4.43	3.42
**Chain C**	2.91	2.20	4.25	3.36	3.18
**Chain D**	3.39	2.38	3.93	3.44	3.28

**Table 2:** RMSD values between the best frames extracted from the MD simulation and the three crystal structures of TRPM8, considering the four chains. The RMSD values are reported in Å and refer both to the backbone atom only and all the heavy atoms of the binding site residues.

	Binding Site	6NR2		6NR3	
		Frame	RMSD	Frame	RMSD
**Chain A**	Backbone	566	3.27	999	2.71
	Side chains	1047	3.81	1000	3.23
**Chain B**	Backbone	990	2.70	991	2.77
	Side chains	1049	3.72	1049	3.74
**Chain C**	Backbone	562	2.09	715	2.25
	Side chains	562	3.20	655	3.39
**Chain D**	Backbone	553	2.58	1246	2.27
	Side chains	900	3.22	1244	3.29

**Table 3:** RMSD values between the poses computed by the compared docking programs within the selected frames and the corresponding poses in the resolved complexes. For docking simulations, the selected frames were utilized before (3A) and after (3B) protein minimization by considering all the four chains. The results by Glide are reported only in Table 3B because it minimizes by default the protein structure. The RMSD values are reported in Å and the best values are in bold.

Table 3A	Frame	PLANTS ChemPLP	GOLD ChemPLP	GOLD GoldScore	GOLD ASP		LiGen™
**6NR2 WS-12**							
**Chain A**	566	7.62	7.96	8.18	7.83		3.55
	1047	8.07	9.07	8.24	8.58		3.35
**Chain B**	990	9.63	7.51	7.50	5.12		3.51
	1049	8.30	7.71	7.17	7.96		3.31
**Chain C**	**562**	7.88	**3.54**	**3.59**	4.42		**3.79**
**Chain D**	553	11.25	8.45	9.09	8.41		3.75
	900	11.86	11.30	11.46	12.01		nn
**6NR3 Icilin**							
**Chain A**	999	6.51	7.04	7.84	7.28		6.85
	1000	7.20	6.93	8.20	7.07		7.08
**Chain B**	991	8.20	6.54	7.96	8.93		3.30
	**1049**	9.10	**3.71**	8.33	**3.18**		**3.06**
**Chain C**	715	12.31	12.37	5.12	5.12		7.34
	655	4.78	8.08	8.33	6.01		4.77
**Chain D**	1246	14.06	7.64	4.28	7.91		nn
	1244	14.75	4.78	4.77	5.06		nn
**Table 3B**	**Frame**	**PLANTS ChemPLP**	**GOLD ChemPLP**	**GOLD GoldScore**	**GOLD ASP**	**LiGen**™	**Glide**
**6NR2 WS-12**							
**Chain A**	566	7.99	4.92	5.25	7.80	3.82	4.87
	1047	5.73	9.00	6.03	8.07	7.61	5.58
**Chain B**	**990**	8.99	**2.69**	**2.90**	9.90	7.11	6.26
	1049	8.05	8.42	6.04	8.30	2.88	7.87
**Chain C**	562	7.73	7.53	7.74	4.31	7.69	8.95
	562	7.73	7.53	7.74	4.31	7.69	8.95
**Chain D**	553	11.27	8.20	10.83	8.52	3.56	6.13
	900	12.41	8.52	8.38	8.42	nn	nn
**6NR3 Icilin**										
**Chain A**	999	8.00	7.54	8.08	7.28	6.90	7.35
	1000	6.68	8.86	8.19	7.89	6.95	5.63
**Chain B**	991	5.30	7.65	7.34	8.07	7.61	7.38
	1049	5.95	7.75	7.90	7.68	3.11	6.74
**Chain C**	715	11.37	5.42	5.03	11.86	2.25	8.44
	655	8.22	8.99	8.46	8.04	4.70	8.52
**Chain D**	1246	13.89	4.97	4.59	5.13	nn	nn
	1244	11.89	4.98	6.72	5.28	nn	nn

**Table 4:** Best EF 1% values plus the corresponding averages as computed considering separately each monomer and each frame and using the three selected programs. The reported EF 1% values refer to the best single primary scoring functions as well as the best models generated by EFO including also the results coming from the rescoring calculations.

Monomer	LiGen™ 1 score	LiGen™ EFO	Gold 1 score	Gold EFO	Plants 1 score	Plants EFO	Mean 1 score	Mean EFO
**562 (overall mean = 10.23)**								
**Chain A**	5.76	34.54	0.00	9.59	5.76	30.71	3.84	24.95
**Chain B**	3.84	26.87	0.00	1.92	0.00	5.76	1.28	11.52
**Chain C**	11.51	28.79	0.00	1.92	1.92	13.43	4.48	14.71
**Chain D**	3.84	40.30	1.92	5.75	0.00	11.51	1.92	19.19
**Mean**	6.24	32.62	0.48	4.80	1.92	15.35	2.88	17.59
**990 (overall mean = 9.16)**								
**Chain A**	15.35	65.25	13.42	13.42	5.76	11.54	11.51	30.07
**Chain B**	9.60	17.27	0.00	1.92	0.00	1.92	3.20	7.04
**Chain C**	3.84	17.27	0.00	1.00	1.92	9.60	1.92	9.29
**Chain D**	3.84	21.11	0.00	3.84	0.00	1.92	1.28	8.96
**Mean**	8.16	30.23	3.36	5.05	1.92	6.25	4.48	13.84
**1049 (overall mean = 18.43)**								
**Chain A**	26.87	57.57	1.92	1.92	7.68	38.38	12.16	32.62
**Chain B**	5.76	40.30	0.00	3.84	1.92	19.19	2.56	21.11
**Chain C**	13.43	28.79	9.62	9.59	3.84	26.87	8.96	21.75
**Chain D**	53.73	55.65	0.00	1.00	1.92	32.62	18.55	29.76
**Mean**	24.95	45.58	2.89	4.09	3.84	29.27	10.56	26.31
**Global Mean**	13.11	36.14	2.24	4.64	2.56	16.95	5.97	19.25

**Table 5:** Best consensus models and the corresponding EF 1% values as generated by the EFO approach and combining the docking results of the four monomers of each frame (Equations 1−6) as well as the four monomers of all three frames (Equations 7−14). In the name of the included scores, the suffixes indicate the involved chain and frame.

Equation	Frame	Docking Tool	Model	EF 1%
1	562	LiGen™	1.00 PS_D – 2.39 MLPINS_A + 19.76 PLP_A – 13.08 MLPINS_C	46.15
2	562	PLANTS	-1.00 MLPINS_A – 3.02 PLP95_NORM_HEVATMS__C	34.62
3	990	LiGen™	-1.00 MLPINS_B + 0.13 CHEMPLP_B + 54.59 Contacts_NORM_HEVATMS__A – 5.39 Xscore_A	59.23
4	990	PLANTS	-1.00 MLPINS_B + 534.45 Contacts_NORM_HEVATMS__A – 14.77 PLP95_NORM_HEVATMS__A	32.69
5	1049	LiGen™	1.00 Contacts_A – 14.20 PLP95_NORM_HEVATMS__A – 13.66 MLPINS_C – 5.35 Csopt_D	57.69
6	1049	PLANTS	1.00 Contacts_NORM_WEIGHT__C – 0.0092 MLPINS_D + 0.041 MLPINS_D – 0.10 PLP95_NORM_HEVATMS__A	48.08
7	All	PLANTS (1)	MLPINS_A_562	23.08
8	All	PLANTS (2)	-1.00 MLPINS_A_562 – 4.59 Xscore_HM__D_1049	38.38
9	All	PLANTS (3)	-1.00 MLPINS_A_562 + 5.34 MLPINS_C_1049 + 50.34 Contacts_NORM_HEVATMS__D_1049	49.89
10	All	PLANTS (4)	-1.00 MLPINS_A_562 + 33.10 Contacts_NORM_HEVATMS__D_562 + 4,33 MLPINS_C_1049 – 0,12 MLPINS_C_1049	51.82
11	All	LiGen™ (1)	Contacts_NORM_HEVATMS__A_990	38.38
12	All	LiGen™ (2)	1.00 PS_D_562 + 0.46 Contacts_NORM_HEVATMS__A_990	55.65
13	All	LiGen™ (3)	-100 MLPINS_B_990 + 0.84 Contacts_A_990 – 1.84 CS_D_1049	65.28
14	All	LiGen™ (4)	1.00 Contacts_NORM_WEIGHT__D_990 – 0.042 MLPINS_B_990 + 0.053 Contacts_A_990 – 0.031 Csopt_D_1049	67.11

## Abbreviations

TRPM8	Transient Receptor Potential Cation Channel Subfamily M (Melastatin) 8
PIP2	Phosphatidylinositol 4,5-Bisphosphate
MD	Molecular Dynamics
VS	Virtual Screening
EFO	Enrichment Factor Optimization
RMSD	Root-Mean-Square Deviation
EF	Enrichment Factor
MLPInS MRC	Molecular Lipophilicity Potential Interaction Score Multiple Receptor Conformations
